# The Effect of Classroom-Based Interventions on Sedentary Behavior and Spinal Health in Schoolchildren: Systematic Review

**DOI:** 10.2196/39006

**Published:** 2022-10-26

**Authors:** Dominic Fisher, Quinette Louw

**Affiliations:** 1 Division of Physiotherapy Department of Health and Rehabilitation Sciences Stellenbosch University Cape Town South Africa

**Keywords:** sedentary behavior, classroom sitting, spinal health

## Abstract

**Background:**

Multifaceted school-based interventions involving many stakeholders show promise toward the reduction of sedentary behavior (SB) and improved musculoskeletal conditions in schoolchildren. In resource-limited contexts, where schools face multiple, complex demands, broad school-based interventions may not be possible. In these settings, less complex, resource-efficient interventions are more likely to be adopted and implemented. Interventions that are limited to classrooms and that do not require broader stakeholder participation may be more appropriate to lower-resource settings.

**Objective:**

The aim of this study was to systematically search for, identify, and summarize the literature on the effectiveness of classroom-based interventions on SB and spinal health in schoolchildren.

**Methods:**

PubMed, EBSCOhost CINAHL, Web of Science, and Scopus were searched between January 1, 2021, and April 30, 2021. We included experimental studies conducted exclusively in school classrooms that objectively measured classroom SB and spinal health. The search terms related to SB, classroom sitting, and classroom neck and back pain. Studies that reported on objectively measured classroom physical activity and instrumented observation of healthy spinal behavior were included in the review. The included studies were critically appraised using the McMaster critical review form for quantitative studies. The study findings were summarized in tables, and a meta-analysis of homogeneous review outcome data was conducted.

**Results:**

Overall, 12 experimental studies from high-income countries were included: 9 (75%) studies focused on SB, and 3 (25%) focused on spinal health. Of the 9 SB studies, 8 (89%) reported decreases in classroom sitting time. The pooled medium-term effects of a subset of SB interventions showed statistically significant decreases in sitting time (*P*=.03), whereas short-term effects and long-term effects were not significantly reduced (*P*=.13 and *P*=.23, respectively). A meta-analysis of spinal health studies demonstrated statistically significant improvements in spinal behavior during functional tasks (*P*=.005).

**Conclusions:**

Classroom-based interventions aimed at reducing SB and improving spinal health may be effective without placing an additional burden on teachers and parents. SB interventions must include strategies to overcome teachers’ and learners’ hedonic motivation to sit during class time. Standardized outcomes for school-based SB are encouraged so that findings from various settings may be pooled to determine the overall effect across studies. The use of standardized functional outcomes in spinal health studies will aid in determining the effectiveness of spinal health interventions across studies.

**Trial Registration:**

PROSPERO International Prospective Register of Systematic Reviews CRD42020176080; https://www.crd.york.ac.uk/prospero/display_record.php?ID=CRD42020176080

## Introduction

### Background

Globally, noncommunicable disorders such as musculoskeletal conditions (eg, low back pain [LBP] and neck pain) and cardiovascular diseases (eg, stroke) are a growing cause of disability [1]. Furthermore, the need for rehabilitation services for these kinds of conditions has increased in inverse proportion to countries’ income levels [2]. The prevention of noncommunicable diseases, especially in regions burdened by infectious diseases such as HIV and AIDS and tuberculosis, is an important health strategy [3]. Although the causes of back pain and cardiovascular disease are multifaceted, rehabilitation professionals have focused on the relationship between these health burdens and sedentary behavior (SB). The Sedentary Behavior Research Network defines SB as “any waking behavior characterized by an energy expenditure ≤1.5 METS [metabolic equivalents] while in a sitting or reclining posture” [4]. Epidemiological associations between SB and a range of noncommunicable diseases such as type 2 diabetes, obesity, spinal musculoskeletal injury, and even some cancers are corroborated by physiological evidence [5]. Redundant understanding (understanding that has been informed by new knowledge and is no longer useful or current) of the physiology of SB encouraged researchers to investigate remedies for the effects of SB with interventions aimed at increasing moderate to vigorous physical activity [6]. The realization that the effects of SB are not “equally and oppositely matched” by the benefits of moderate to vigorous physical activity has prompted researchers to instead trial interventions to reduce the accumulation of SB [6]. This preventive approach to addressing sedentary behavioral physiology is germane to recommended preventive measures of sitting-related back pain.

The causal relationship between SB (such as sitting) and the onset of back pain is complex, as is apparent in the contradictory findings in the literature [7]. Although the methodological weakness of studies may account for some of these equivocal findings, another important factor may be the heterogeneity of back pain. The effects of (particularly prolonged) static sitting on the structures of the spine include continuous intervertebral disk compression and resultant compromised disk nutrition. Furthermore, in vitro studies have demonstrated how intervertebral disk tissue deforms under loads comparable to the compressive loads experienced during sitting [8]. In the absence of an optimal sitting posture [7], a proposed strategy to mitigate the effects of prolonged sitting on the spinal structures is dynamic sitting [9]. The rationale of this strategy is to encourage small-range high-frequency changes in the spine, using specific chairs or equipment, to reduce continuous loading of spinal structures [9]. However, the efficacy of dynamic sitting is limited. As such, researchers turned to assessing the efficacy of strategies aimed at reducing total sitting time, particularly by breaking up prolonged periods of sitting, as a means of mitigating the effects of sitting on the spinal tissues [10]. Interrupting prolonged periods of sitting with alternative postures such as standing is purported to balance the load on musculoskeletal tissues, mitigating the onset of soft tissue strain and delaying the onset of discomfort.

Reducing the accumulation of SB by interrupting prolonged periods of sitting has also been suggested as a means of arresting harmful SB physiology [11,12]. Given that SB continues from childhood into adulthood [13], much scientific research on SB in children has been published in the last decade [14]. SB in children has consistently been associated with increased cardiometabolic disease, including insulin resistance [15] and decreased high-density lipoprotein cholesterol [16]. Developing effective preventive strategies that are designed to limit cardiometabolic health problems associated with SB during childhood seems prudent considering its effect on health-related quality of life [17,18] across the life span.

Considering that LBP also tracks across the life span from adolescence into adulthood [19] and given the association between SB and LBP, early interventions aimed at preventing SB from becoming ingrained at school-going age has the potential to address manifold health burdens. The World Health Organization has encouraged the coordination of health and education systems in health promotion for several decades [20]. SB is ubiquitous during school time, with class time being the most sedentary period [21]. The past decade has seen many studies published on strategies aimed at reducing SB in schools [22].

Hegarty et al [23], in their review of school-based studies aimed at reducing SB in children, describe the range of underpinning theoretical bases. Although the design of interventions based on various theoretical underpinnings is justified, the pragmatic challenges inherent to resource-constrained contexts hinder the feasibility of interventions that require additional resources beyond the status quo. Interventions underpinned by social cognitive theory or social frameworks [24] that burden teachers with teaching SB-related curricula in addition to the normal academic content may stretch a school’s human resources to the point that the intervention becomes unfeasible [25]. Furthermore, interventions that require parents to engage with learning materials [26] are not feasible in contexts of low adult literacy, low parent-child engagement, and prevalent child-headed families, as are common in low-income countries plagued by war or epidemics. According to the capability, opportunity, and motivation behavior framework [27], school-based interventions that involve changes to the physical environment of the classroom (opportunity) and that neither depend on participants’ acquisition of additional capability nor increase participants’ motivation for SB are likely to succeed in reducing classroom SB [28].

### Objectives

In resource-constrained contexts, it is important that public health programs are underpinned by sound theoretical frameworks that increase the likelihood of succeeding and that address multiple health burdens. Given the potential benefits of reducing SB in relation to noncommunicable diseases and spinal health across the life span, reviewing the literature on the implementation of classroom-based interventions aimed at reducing SB and improving spinal health will provide important information in deciding what strategies to implement in the South African context. This review, which aimed to identify and summarize evidence on the effectiveness of classroom-based interventions aimed at reducing SB and improving spinal health, certainly meets these criteria.

## Methods

This systematic review was registered with PROSPERO in November 2020. The review was conducted according to the PRISMA (Preferred Reporting Items for Systematic Reviews and Meta-Analyses) statement [29].

### Eligibility Criteria

The following inclusion and exclusion criteria were considered for this review.

#### Types of Studies

Given that this review aimed to identify and summarize the effectiveness of interventions, studies with any form of experimental design were included. Studies with experimental designs include case studies and case series, uncontrolled before-and-after trials, interrupted time series trials, nonrandomized controlled trials, cluster randomized controlled trials, and randomized controlled trials. All English-language studies published until April 30, 2021, were considered.

#### Types of Participants

Participants enrolled at primary and high school classrooms were included in this review.

#### Types of Interventions

Any classroom-based interventions aimed at reducing classroom SB and improving back health were considered for this review. Examples of classroom-based interventions that were considered included, but were not limited to, education programs, movement integration, exercise or movement programs, or changes to the classroom environment. Only interventions that were conducted within the confines of the classroom were considered.

#### Types of Comparisons

Comparison groups had to be subject to the usual classroom conditions.

#### Types of Outcomes

Studies had to report on objectively measured classroom sitting time, bouts of prolonged periods of sitting, frequency of interruptions to sitting, spinal muscle strength, or instrumented observation of healthy spinal behavior.

#### Exclusion Criteria

Observational studies were not considered for inclusion in this review because they would not be able to infer the effectiveness of interventions. Studies with participants who required special education and mobility needs were excluded because these school classrooms are often adapted to facilitate the educational and mobility requirements of these learners and differ from mainstream school classrooms. In addition, studies that included participants with spinal pain related to injury or disease were not eligible for inclusion. Experimental studies with intervention strategies with components that go beyond the school classroom or school time were also not considered eligible for inclusion. Studies that did not apply the definition of SB as behavior that expends ≤1.5 metabolic equivalents [4] were excluded.

### Search Strategy

The following electronic databases were comprehensively searched from database inception to April 30, 2021: PubMed, EBSCOhost CINAHL, Web of Science, and Scopus. Database searches included a combination of Medical Subject Headings (PubMed) and similar keywords in combination with Boolean operators such as *OR* and *AND* according to the database function. An example of the combination of keywords searched included (*school OR class OR classroom*) AND (*sedentary behavior OR sedentariness OR sitting*) for SB studies and (*school OR class OR classroom*) AND (*spine OR spine health OR posture*) for studies on spinal health. Only language filters were applied to searches. One researcher (DF) conducted all searches. Hand searches of reference lists of the included studies to identify additional studies were conducted.

### Study Selection

The results from the 4 database searches were screened by study title and abstract according to the eligibility criteria by one researcher (DF). Duplicate studies were identified, exported to Microsoft Excel, and manually removed. A second researcher (QL) spot-checked the potential included studies for incorrect study retention. Thereafter, retained studies were screened by full-text reading of the papers. The second researcher (QL) repeated the spot-check once full-text screening was completed. Consensus was then reached about the inclusion of studies in the review. Study eligibility was assessed by one researcher (DF), with uncertainties discussed with the second researcher (QL).

Search results from the respective electronic databases were exported to Mendeley reference management software (Mendeley Ltd). The results were copied into a customized Microsoft Excel sheet to document the review results, identify and exclude duplicates, and track information for the PRISMA flowchart [29].

### Methodological Appraisal

The included studies were critically reviewed using the McMaster critical review form for quantitative studies [30]. This review tool allows the researcher to appraise the included studies on the stated purpose of the study and relevance of the background literature, appropriateness of the study design, study sample and selection, measurement and detection bias, sample size, outcomes and results, and conclusion and implications. Studies were not excluded based on quality.

### Data Extraction

Data from the included studies were extracted according to the study design, participant information, intervention description, study outcomes, and intervention effects. The information was recorded in a customized Microsoft Excel data extraction form by the first author (DF). The second author (QL) performed a spot-check on a subsample of the included studies to assess accuracy and consistency. Consensus about the extracted data was reached between the researchers before data synthesis. Data was extracted according to the following categories:

- General study information: date of extraction, author or authors, title, type of publication, and country of origin
- Study characteristics: study aims and objectives and study design
- Participants: population and setting and number of participants
- Intervention features: intervention setting and mode of intervention
- Measurement description: unit of measurement, type of measurement, frequency, and follow-up duration

### Data Analysis

The key outcomes were SB and spinal health. We used Review Manager (version 5.4.1; The Cochrane Collaboration) [31] to conduct a meta-analysis of SB and spinal health outcomes where required data (mean and SD or SE) were available, and outcomes were similar. The data to conduct a meta-analysis were extracted from published manuscripts and supplementary files, requested from the corresponding author or derived using the calculator function in Review Manager. A random effects model for heterogenous data among studies was used. We conducted a subgroup analysis based on the follow-up period in different studies (short: <12 weeks, medium: 12-24 weeks, or long term: ≥24 weeks). The overall measure of effect (mean difference and 95% CIs) was calculated for all outcomes and subgroups, and we considered the *I*^2^ test as the measure of subgroup heterogeneity. Effects and *P* values of SB and spinal health outcomes that were too dissimilar and not appropriate for inclusion in the meta-analysis were calculated using MedCalc (version 20.113; MedCalc Software Ltd) [32] and tabulated.

## Results

### Study Selection

**Figure 1:**
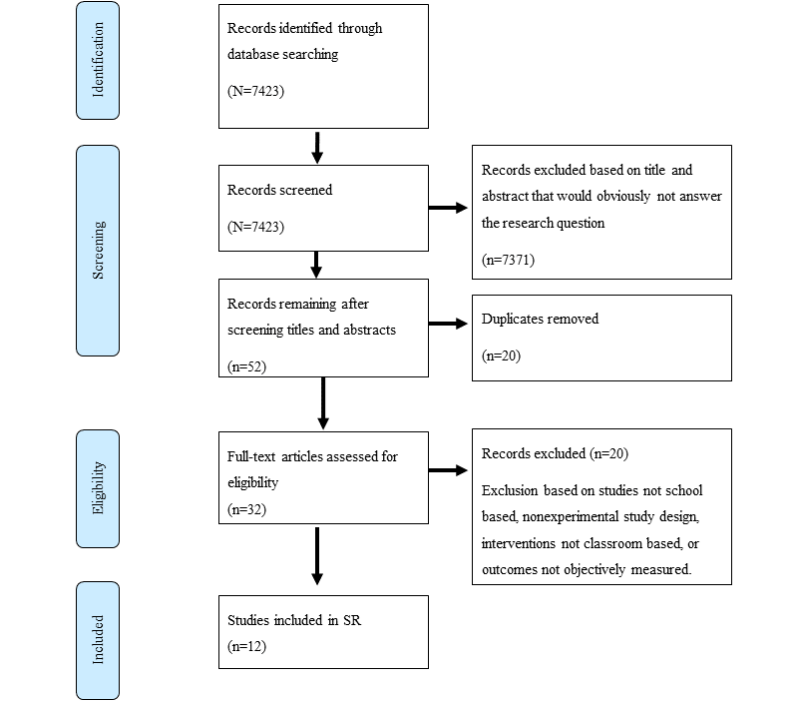
PRISMA (Preferred Reporting Items for Systematic Reviews and Meta-Analyses) flow diagram of search results and included studies. SR: systematic review.

The search results from the 4 databases used yielded 7423 studies. A total of 12 papers met the inclusion criteria, each reporting on individual studies, and were included in the review (Figure 1).

### Study Characteristics

#### Participants and Study Location

The included studies comprised 2296 participants: 1026 (44.69%) in SB studies and 1270 (55.31%) in spinal health studies (Table 1). Study sample sizes ranged from 23 to 696 participants with ages ranging from 8 to 17 years. Of the 12 studies, 2 (17%) had male-only participants [33,34], whereas the remaining studies (10/12, 83%) had male and female participants. Of the 12 studies, 6 (50%) had >100 participants, and 1 (8%) had >500 participants.

**Table 1:** Characteristics of included studies.

Country (setting), study			Study aim			Study design (sample size)		Sample description (age [years])
**Sedentary behavior studies**								
	United Kingdom (2 primary school classes), Sherry et al [35]	To assess the impact of a full standing desk allocation system on sitting behavior and to explore changes in behavior-related mental health, MSK^a^ health, and markers of cognitive function			Pilot controlled trial (n=55)		Year 5 students (9-11)	
	Australia (2 primary school classes), Contardo Ayala et al [36]	To assess the impact of an intervention incorporating height-adjustable desks and pedagogical strategies on overall volume and pattern of classroom sitting			Pilot nonrandomized trial (n=41)		Year 6 students (11-12)	
	Belgium (10 primary and 9 secondary schools), Verloigne et al [37]	To conduct an effect evaluation of implementing standing desks in the classroom and evaluate the process of implementing standing desks			Cluster RCT^b^ (n=343)		Grade 5 students (10-11) and grade 10 students (15-16)	
	Portugal (1 primary school), Silva et al [38]	To investigate the impacts of a classroom standing desk intervention on classroom sitting time and verify effects of the intervention on whole-day SB^c^ and PA^d^ during the week and weekend			Cluster controlled trial (n=49)		Grade 6 students (11-13)	
	United Kingdom (10 primary schools), Norris et al [39]	To test the effect of a PA class intervention on children’s PA and SB, on-task behavior, and student engagement			Cluster RCT (n=264)		Year 4 students (8-9)	
	Australia (2 primary schools), Ee et al [34]	To determine the effects of a classroom standing desk intervention on school sitting and standing time, waking hours PA and SB, and MSK discomfort			Within-participants crossover trial (n=47)		Grade 4 students (10-11); only male participants	
	Australia (1 secondary school), Sudholz et al [40]	To examine the impact of combining environmental change and classroom prompts on adolescents’ classroom sitting time, prolonged sitting bouts, standing and stepping time, and sitting interruptions			Quasi-experimental design (n=105)		Grades 7, 10, and 11 students (12-17)	
	Australia (1 primary school), Parry et al [33]	To assess effects of yearlong intermittent use of a standing desk on sitting and standing time at school and sedentary time and PA for waking hours, as well as self-reported presence and intensity of MSK symptoms			Repeated measures within-participants crossover trial (n=23)		Grade 4 students (9-10); only male participants	
	United States (9 elementary schools), Swartz et al [41]	To determine the effect of stand-biased desks on PA and SB in elementary schoolchildren and examine the impact of stand-biased vs sitting desks on SB and activity during the school day			Within-classroom crossover design (n=99)		Grades 3, 4, and 6 students; age not reported	
**Spinal health studies**								
	Belgium (3 primary Schools), Cardon et al [42]			To investigate the efficacy of a back education program and examine habit changes	RCT (n=696)		Grades 4 and 5 students (9-11)	
	Germany (2 primary schools), Dullien et al [43]			To examine whether teacher-led intervention programs could improve back-care knowledge, back-friendly behavior, and core muscle endurance	Cluster RCT (n=176)		Grade 5 students (10-12)	
	Belgium (8 elementary schools), Geldhof et al [44]			To investigate the effect of an optimized multifactorial back education program on knowledge and postural behavior in children	Quasi-experimental pre-post design (n=398)		Elementary school students (9-11)	

^a^MSK: musculoskeletal.

^b^RCT: randomized controlled trial.

^c^SB: sedentary behavior.

^d^PA: physical activity.

The 12 included studies were all conducted in high-income regions, namely Europe (n=5, 42%) [37,38,42-44], Australia (n=4, 33%) [33,34,36,40], the United Kingdom (n=2, 17%) [35,39], and the United States (n=1, 8%) [41]. Of the 12 studies, 9 (75%) focused on the intervention’s effects on SB [33-41], and 3 (25%) focused on spinal health [42-44]. Of the 12 studies, 10 (83%) were conducted in primary schools [30,33-38,40,44], 1 (8%) in a secondary school [40], and 1 (8%) in both primary and secondary schools [37].

#### Description of Interventions

Of the 9 SB study interventions, 1 (11%) [39] used physically active lessons (Table 2). The remaining SB study interventions comprised either adding to [37], replacing all [35,36,38,40], or replacing a proportion of the traditional classroom desks with stand-biased desks [33,34,41]. Of the 9 studies, 5 (56%) [35-38,40] included teacher training and development as part of the intervention. All the spinal health studies used a back education program [42-44]. Of the 3 studies, 2 (67%) [43,44] added posture awareness training to the back education program, and 1 (33%) [43] included an exercise component in the intervention.

**Table 2:** Summary of study interventions.

Study		Intervention description			Intervention duration			Theoretical underpinning		
**Sedentary behavior studies**										
	Sherry et al [35], 2020		All usual desks replaced with height-adjustable sit-stand desks. Instructional posters in classroom demonstrating correct posture, environmental change to classroom, reflective motivation from teacher, and monthly visits from researchers			8 months			Behavior change wheel [45]; COM-B^a^ model	
	Contardo Ayala et al [36], 2016	All usual desks replaced with height-adjustable sit-stand desks and original chairs replaced with laboratory stools; teacher development about pedagogical approaches to reduce and break sitting and how to adapt delivery of usual curriculum and safe use of desk			8 months			Not reported		
	Verloigne et al [37], 2018	Three standing desks introduced into class, and teachers received presentation to situate the intervention in health context based on evidence (printed presentation material provided to teachers)			6 months			Not reported		
	Silva et al [38], 2018	Traditional seated desks exchanged for adjustable sit-stand standing desks, teacher training sessions by physical education and psychology professionals, and family support sessions			16 weeks			Not reported		
	Norris et al [39], 2018	Physically active lesson intervention “Virtual Traveler” comprised three 10-minute physically active sessions per week (18 sessions)			6 weeks			BCTTv1^b^ [46]		
	Ee et al [34], 2018	Class divided in half and rotated through use of height-adjustable stand-up desks, whereas other half used traditional desks on 21-day cycle			6 weeks			Not reported		
	Sudholz et al [40], 2020	Traditional classroom desks exchanged for height-adjustable desks and backless laboratory stools; 3 posters and desk stickers to provide behavioral prompts; and 1-hour teacher training on how to use desks, evidence of health benefits of breaking up sitting time, and tips and strategies for adolescents			6 months			Not reported		
	Parry et al [33], 2019	Class divided in half and rotated through use of standing desks for 21-day cycle throughout school year			8 months			Not reported		
	Swartz et al [41], 2019	Half the class allocated stand-biased desk for 9 weeks before using sitting desk for 9 weeks; other half allocated sitting desk for 9 weeks before using stand-biased desk for 9 weeks			18 weeks			Not reported		
**Spinal health studies**										
	Cardon et al [42], 2002			Six weekly 60-minute back education sessions delivered by physical therapist based on biomechanical literature and the German program of back exercises. The program made use of 10 “make your disks happy” guidelines			6 weeks			Not reported
	Dullien et al [43], 2018			Teacher-delivered 5 back-care lessons (materials provided) focusing on anatomical knowledge of the spine, good and bad sitting posture, healthy backpack habits and lifting, healthy carrying, and back-friendly sport and nutrition; posture awareness training and improvement posters put up in classroom; and mandatory back and abdominal muscle exercises at the beginning of each class			1 school year (10 months)			Not reported
	Geldhof et al [44], 2006			Six weekly back education lessons by physical therapist included back anatomy and pathology as well as principles of biomechanical postures during standing, sitting, lying, lifting, pushing, and bending; 10 large posters of back posture principles; and stimulation of dynamic sitting by introducing 2 Pezzi balls and a Dynair wedge for each classroom, as well as twice daily movement breaks			2 years			Not reported

^a^COM-B: capability, opportunity, and motivation behavior.

^b^BCTTv1: Behavior Change Technique Taxonomy version 1.

### Critical Review of Included Studies

Table 3 provides a summary of the McMaster critical review [47] of the included studies. All included papers clearly stated the study purpose, adequately described the sample, and provided sufficient details regarding the intervention. Furthermore, all studies reported the study results in terms of statistical significance, used appropriate methods of analysis, and reported on the clinical importance of their results. Of the 12 studies, 11 (92%) provided a justification for the sample size, whereas 1 (8%) [39] did not. The spinal health studies [42-44] all showed weakness in terms of validity of the outcome measures used and lack of control of contamination as part of their design.

**Table 3:** McMaster critical review form for quantitative studies.

			Sedentary behavior studies																			Spinal health studies				
			[35]		[36]		[37]		[38]		[39]		[34]		[40]		[33]		[41]		[42]			[43]		[44]
Study purpose: was the purpose clearly stated?			✓		✓		✓		✓		✓		✓		✓		✓		✓		✓			✓		✓
Literature: was relevant background literature reviewed?			✓		✓		✓		✓		✓		✓		✓		✓		✓		✓					✓
**Sample**																										
	Was the sample described in detail?	✓		✓		✓		✓		✓		✓		✓		✓		✓		✓					✓	
	Was the sample size justified?									✓																
**Outcomes**																										
	Were the outcome measures reliable?	NR^a^		✓		NR		✓		✓		✓		✓		✓		✓								
	Were the outcome measures valid?	NR		✓		✓		✓		✓		✓		✓		✓		✓								
**Intervention**																										
	Was the intervention described in detail?	✓		✓		✓		✓		✓		✓		✓		✓		✓		✓			✓		✓	
	Was contamination avoided?	✓				✓				✓		N/A^b^				N/A		N/A		✓					✓	
**Results**																										
	Were the results reported in terms of statistical significance?	✓		✓		✓		✓		✓		✓		✓		✓		✓		✓			✓		✓	
	Were the analysis method or methods appropriate?	✓		✓		✓		✓		✓		✓		✓		✓		✓		✓					✓	
	Was the clinical importance reported?	✓		✓		✓		✓		✓				✓		✓										
	Were dropouts reported?	✓				✓				✓		✓						✓		✓					✓	
Conclusions and implications: were the conclusions appropriate given the study methods and results?					✓		✓		✓		✓		✓		✓		✓		✓		✓					✓

^a^NR: not reported.

^b^N/A: not applicable.

### Variability of Study Outcomes in SB Studies

The SB studies reported outcomes either in relation to school time [33,34,37,38,41] or class time [35,36,39,40] (Table 4). There were also differences in the units of measurement used, namely minutes [35-37,39], minutes per 9 hours [33,38], minutes per day [34,41], and minutes per lesson [40]. Sitting was the main proxy measure used for SB, but 25% (3/12) of the studies included proxy measure variations such as frequency of 30-minute sitting bouts; sitting time accumulated in >5-minute, 10-minute, and 20-minute sitting bouts; and sitting time accumulated in >15-minute bouts [44].

**Table 4:** Intervention effects on sedentary behavior and spinal health in included studies.

Study		Measure of effect (units)		Value			*P* value	Direction of the effect
**Sedentary behavior studies**								
	Sherry et al [35], 2020	Mean difference (95% CI) sitting time as percentage of wear time				–25.34 (–32.25 to –18.43)^a^; –19.99 (–27.05 to 12.94)^b^	.001^a^; .008^b^	Improved^a^; improved^b^
	**Contardo Ayala et al [36], 2016**							
		Mean difference (95% CI) classroom time sitting bouts >5 minutes				–10.4 (–25.76 to 4.96)^b^	.19	No effect
		Mean difference (95% CI) classroom time sitting bouts >10 minutes				–17.67 (–33.78 to 1.56)^b^	.03	Improved
		Mean difference (95% CI) classroom time sitting bouts >20 minutes				–10.21 (–26.72 to 6.31)^b^	.23	No effect
	**Verloigne et al [37], 2018**							
		Mean difference (SE) school hours frequency of sitting bouts ≥30 minutes				Primary school –0.578 (0.364)^c^; secondary school 0.463 (0.545)^c^	>.10^c^; >.10^c^	No effect^c^; no effect^c^
		Mean difference (SE) school hours time accumulated in sitting bouts ≥30 minutes				Primary school –30.518 (17.245)^c^; secondary school 26.073 (26.802)^c^	≥.05 to .10^c^; >.10^c^	No effect^c^; no effect^c^
	Sudholz et al [40], 2020	Mean difference (95% CI) sitting in >15-minute bouts (minutes per lesson)				–7.7 (–17.5 to 2.0)^c^; –11.2 (–18.0 to –4.5)^a^	.14^c^; .002^a^	No effect^c^; improved^a^
	**Swartz et al [41], 2019**							
			Mean difference (95% CI) SB^d^ (minutes per day) grade 3 across-group comparison		12.9 (4.33 to 21.47)^c^; 19.3 (12.64 to 25.96)^a^		.003^c^; <.005^a^	Worsened^c^; worsened^a^
			Mean difference (95% CI) SB (minutes per day) grade 4 across-group comparison		12.4 (0.64 to 24.16)^c^; 4.3 (– 5.73 to 14.33)^a^		.03^c^; .37^a^	Worsened^c^; no effect^a^
			Mean difference (95% CI) SB (minutes per day) grade 6 across-group comparison		4.2 (– 10.61 to 19.01)^c^; –14.4 (–28.69 to – 0.11)^a^		.57^c^; .04^a^	No effect^c^; improved^a^
**Spinal health studies**								
	**Cardon et al [42], 2002**							
		Mean difference (95% CI) practical test score		19.47 (16.4 to 22.54)^c^; 19.20 (16.96 to 22.88)^a^			<.001^c^; <.001^a^	Improved^c^; improved^a^
		Mean difference (95% CI) candid camera score		8.23 (5.96 to 10.58)^b^			≤.001	Improved
		Percentage change weekly back or neck pain prevalence		–5.1^c^; –7.9^a^; –8.6^b^			Not reported^c^; not reported^a^; <.05^b^	Not reported^c^; not reported^a^; improved^b^
	**Dullien et al [43], 2018**							
		Percentage reduced back pain frequency		–25.89^b^			.84	No effect
		Mean difference (95% CI) back knowledge score		2.6 (1.5 to 3.7)^b^			.001	Improved
	**Geldhof et al [44], 2006**							
		Mean difference (95% CI) general back posture knowledge		2.4 (1.75 to 3.05)^b^			*<*.001	Improved
		Mean difference (95% CI) specific back posture knowledge		1.2 (0.23 to 2.17)^b^			*<*.001	Improved
		Mean difference (95% CI) percentage lesson duration static sitting		5.7 (–6.72 to 18.12)^b^			Not reported	Not reported
		Mean difference (95% CI) percentage lesson duration dynamic sitting		1.9 (–1.93 to 5.73)^b^			Not reported	Not reported
		Mean difference (95% CI) percentage lesson duration trunk flexion		–7.4 (–22.80 to 8.00)^b^			<.05	Improved
		Mean difference (95% CI) percentage lesson duration trunk torsion		–0.60 (–4.9 to 3.7)^b^			.79	No effect
		Mean difference (95% CI) percentage lesson duration neck flexion		30.3 (–4.92 to 11.52)^b^			Not reported	Not reported
		Mean difference (95% CI) percentage lesson duration neck torsion		–0.60 (–4.90 to 3.70)^b^			<.05	Improved

^a^Measurement period: <24 weeks.

^b^Measurement period: ≥24 weeks.

^c^Measurement period: <12 weeks.

^d^SB: sedentary behavior.

### Intervention Effects

**Figure 2:**
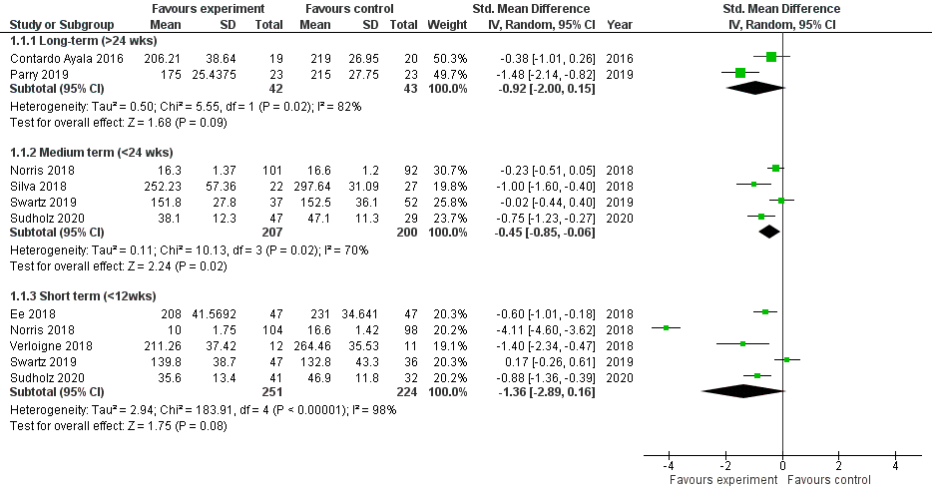
Forest plot of random effects of classroom sitting between intervention and control groups.

The SB study by Swartz et al [41] reported an increase in SB in the intervention group at final follow-up (Figure 2). All the other studies [33-40] reported decreased sitting time, with 75% (6/8) of these studies [33,34,37-40] reporting statistically significant differences. Statistically significant short-term SB intervention effects were reported in 44% (4/9) of the studies [34,37,39,40], whereas 22% (2/9) of the studies [38,40] reported statistically significant intervention effects in the medium term. The study by Parry et al [33] reported statistically significant long-term intervention effects to reduce SB, whereas the study by Sudholz et al [40] reported statistically significant short- and medium-term intervention effects.

**Figure 3:**
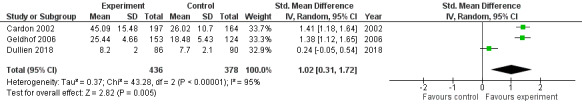
Forest plot of random effects on spinal behavior during functional task between intervention and control groups.

All 3 spinal health studies [42-44] showed statistically significant improvements in spinal behavior during practical or functional tasks; for example, during the practical test [42], carrying a heavy object [43], and during material handling [44] (Table 4). A random effects model analysis of the long-term effects of the spinal health interventions showed a large pooled effect in favor of back health interventions (Figure 3). Both studies that evaluated the intervention effects on back-care knowledge reported statistically significant long-term improvements [43,44]. Of the 2 spinal health studies [42,44] reporting on weekly spinal pain prevalence, 1 (50%) [42] showed a statistically significant decrease in the long term. A statistically nonsignificant reduction in frequency of spinal pain was reported by Dullien et al [43].

## Discussion

### Principal Findings

Our study summarized the effects of classroom-based interventions targeting two separate but related health outcomes, namely SB and spinal health. The main finding is that classroom-based interventions yielded mixed results for SB outcomes and positive results for spinal health outcomes. The interventions used in the included studies were conducted within the classrooms, thus not requiring additional school and community resources.

In our study, it was found that SB interventions mostly aimed to reduce classroom sedentariness by altering classroom behavioral topography (ie, using combinations of teacher and learner education and sit-stand furniture strategies). The exception was the study by Norris et al [39], which used a teacher-led physical activity strategy. Although both strategies yielded positive results in reducing classroom sitting time, the teacher-led physical activity intervention by Norris et al [39] was more effective than the other interventions in the short term (Figure 2). However, the effectiveness tapered off in the medium term, which may imply that teacher-led interventions might not be sustainable after the cessation of the intervention period. The teacher-led physical activity intervention relied on the teacher administering the physical activity and was thus unable to influence the SB of learners outside of the periods in which the activity was being conducted. However, behavioral topography interventions allowed learners to reduce their SB without reliance on the teacher. The interventions based on altering behavioral topography had relatively smaller effects in the short term, but these reductions were maintained at follow-up. The reason for these interventions’ relatively small reduction in SB may be that they either insufficiently addressed teachers’ and learners’ automatic perceptual mechanism to habitually sit during lessons [48] or were unable to alter teachers’ perceptions that they are better able to maintain classroom order and control when learners are seated [49]. The teacher-led physical activity intervention required learners to participate in the physical activity without the need to overcome either of these factors. The mixed findings regarding the effectiveness of interventions on SB contrast with the definitive improvements shown by the spinal health interventions.

Spinal health interventions produced large pooled effects for improving spinal health behavior during functional tasks in the long term (Figure 3). Three distinct methods of intervention were used in the different studies, namely educational lessons [42-44] and accompanying visual aids (such as posters) [43,44], physical activity or exercise programs, and ergonomic devices [43,44]. The intervention by Dullien et al [43] resulted in smaller improvements in spinal health behavior during a functional task than the other spinal health studies. The functional outcome used by Dullien et at [43] consisted of a single carrying task. It is likely that the learners who completed the multidomain functional task used by Cardon et al [42] and Geldhof et al [44] were able to compensate for low scores attained in some of the domains with higher scores achieved in other domains. This was not possible in the single-domain functional task used by Dullien et al [43]. The nonuniformity of this functional outcome across the studies must be considered in the interpretation of this study finding.

The nonuniformity of the reported units of measurement and outcome measurement of studies hindered cross-study comparison. Units of measurement from SB studies included aggregated minutes, minutes per 9 hours, minutes per day, and minutes per lesson. Furthermore, the variability and makeup of spinal health outcome measurements are also problematic; for instance, Cardon et al [42] included sitting posture and ring-binder use at the desk in the observation of practical application of healthy spinal principles, whereas Geldhof et al [44] measured static and dynamic posture separately from other functional tasks. Furthermore, Dullien et al [43] embedded the observation of the demonstration of static and dynamic posture in a back behavioral trial. The use of standard measures of outcome and standardized measurement units is likely to facilitate cross-study comparison and must therefore be addressed by future research in the field.

The heterogeneity in study design across the included studies required the use of a generic critical appraisal tool [47]. The studies were generally well described, which is helpful to researchers planning similar intervention studies. However, the critical review revealed notable shortcomings, namely the nonreporting of clinical importance, lack of a justified sample size, and use of nonvalidated and unreliable outcome measures in the spinal health studies. The lack of reporting of sample size justification in the included studies implies that the studies were underpowered to assess the study outcomes. The use of nonstandardized and unreliable outcome measures may introduce bias into the study findings. These limitations of the included studies undermine the study findings and, by extension, the generalizability of the findings.

The burden posed by spinal health conditions and noncommunicable diseases [50] in low-resource countries matches that in high-income countries. However, the competing demands for resources as well as psychosocial and economic contextual factors in low-income countries must be considered by researchers and program planners intending to conduct similar studies in such countries; for instance, in low-income contexts and settings characterized by marked inequity in society, unequal resource distribution in communities and schools may threaten disproportionate rollout of classroom-based interventions. In addition, the interdepartmental collaboration required to roll out classroom-based health programs at sites managed by departments of education is not guaranteed in resource-scarce settings [51]. Given that all included studies were conducted in high-income countries, the generalizability of our review’s findings may be limited in resource-scarce countries.

A previous systematic review of school-based SB interventions [23] included intervention programs that incorporated strategies that extended beyond the confines of the classroom. These differences are noteworthy because reducing discretionary and nondiscretionary SB may require different intervention strategies [28]. In addition, our study included classroom-based interventions aimed at improving spinal health. The common pedagogical approaches between SB and spinal health studies included in our review (ie, teacher training; the use of an education and training program delivered by the teacher, researchers, or the use of posters; and changing the physical environment of the classroom using alternative classroom furniture or dynamic sitting equipment) are strategies common to both SB and spinal health studies included in our review. Owing to their potential benefits, combining SB and spinal health intervention strategies to create an impact on both health outcomes may be a cost-effective approach for low-resource settings. The commonality between SB and spinal health studies is not surprising given the likely common root problem, namely the accrual of prolonged periods of static sitting in schools [52]. Given the pervasiveness of prolonged classroom sitting and its dual harmful associations with metabolic syndrome and adverse loading patterns of spinal structures, our review provides supportive evidence for the effectiveness of bimodal classroom-based interventions to address both these health outcomes.

All 3 spinal health studies [42-44] included a classroom-based back education program, which included information about the structure and function of the spine during sitting and standing postures as well as functional spinal movement. These back education programs proved effective in producing statistically significant improvement in functional back behavior in the long term. This once more provides evidence for the effectiveness of classroom-based spinal health interventions. Given that both SB and spinal health interventions comprised classroom-based training via teacher-delivered presentations and educational posters or stickers, there may be potential to combine the SB and spinal health messages to address these related health concerns as part of a single, combined intervention strategy. Our study shows promising findings for researchers and health program planners considering implementing school-based interventions that do not require resources beyond the confines of the classroom environment or that may be a burden to the home environment. The relatively small resource footprint of effective classroom-based interventions is preferred in resource-constrained contexts. Programs with limited resource footprint may also improve sustainability [25].

### Strengths and Weaknesses of Included Studies

A strength of our review is the inclusion of objectively reported and measured SB studies, thus eliminating the risk of over- or underestimation owing to participant recall. Another strength is the long-term reporting of spinal health outcomes. A weakness of the SB studies was the variability of the units of measurement, making it difficult to pool the data and conduct a meta-analysis of the intervention effects. High levels of heterogeneity in meta-analysis limits the utility of the study findings. A weakness of the spinal health studies was that although sitting posture and spinal behavior formed part of the back education interventions, intervention effect on sitting posture and behavior made up a small component of the outcome measures used. Furthermore, the functional assessment outcomes were nonstandardized and incongruent with typical classroom behavior. The use of standardized functional assessment outcomes in future studies will improve cross-study comparison.

### Study Limitations

The first limitation of our study pertains to the search strategy used for the different electronic databases. Although we made use of the PubMed database capabilities such as searching using Medical Subject Headings, the full search capabilities of the other 3 databases were not optimized. This may have resulted in missing relevant studies that may have influenced the results of our review and meta-analysis. However, a recent search of the PubMed database yielded results that were similar to those of our initial search. Second, this study is limited by the fact that all the included studies were set in high-income countries. Thus, the findings are not generalizable to low- and middle-income countries. This is particularly pertinent given the relatively expensive SB intervention of height-adjustable sit-stand desks. Third, our study is limited by the inclusion of experimental study designs, which are associated with increased risk of bias. Given the small number of studies found that conducted classroom-based interventions, studies were not excluded based on methodological quality. The need for evidence on the effectiveness of classroom-based interventions in this emerging field was prioritized over the inclusion of studies with less inherent bias.

### Conclusions

The findings of our study suggest that classroom-based interventions may be effective in improving SB and spinal health outcomes without placing a burden on space, equipment, or staff beyond the classroom setting. Our findings show significant effects on spinal health outcomes and positive trends in SB outcomes, although the overall effect was only significant in the medium term. Effective classroom-based interventions can thus potentially be considered by researchers, clinicians, and program planners wanting to develop classroom-based interventions in resource-constrained environments. Future studies to advance interventions aimed at improving SB outcomes must include strategies to overcome teachers’ and learners’ hedonic motivation to sit during class and use appropriate sampling methods and justified sample sizes.

## Abbreviations

LBP: low back pain
PRISMA: Preferred Reporting Items for Systematic Reviews and Meta-Analyses
SB: sedentary behavior
